# Structure and evolution of the mouse pregnancy-specific glycoprotein (*Psg*) gene locus

**DOI:** 10.1186/1471-2164-6-4

**Published:** 2005-01-12

**Authors:** Andrew S McLellan, Beate Fischer, Gabriela Dveksler, Tomomi Hori, Freda Wynne, Melanie Ball, Katsuzumi Okumura, Tom Moore, Wolfgang Zimmermann

**Affiliations:** 1 Department of Biochemistry, Biosciences Institute, University College Cork, College Road, Cork, Ireland; 2 Tumor Immunology Group, LIFE Center, University Clinic Grosshadern, Ludwig-Maximilians-University Muenchen, Marchioninistrasse 23, D-81377 Muenchen, Germany; 3 Laboratory of Molecular and Cellular Biology, Department of Life Sciences, Faculty of Bioresources, Mie University, 1515 Kamihama, Tsu, Mie 514-8507, Japan; 4 Institute of Molecular Medicine and Cell Research, Albert-Ludwigs-University Freiburg, Stefan-Meier-Str. 17, D-97104 Freiburg, Germany; 5 Uniformed Services University of the Health Sciences, 4301 Jones Bridge Road, Bethesda, MD 20814, USA; 6 Division of Rheumatology and Clinical Immunology, Department of Medicine, University Hospital Freiburg, Hugstetter Str. 55, D-79106 Freiburg, Germany

## Abstract

**Background:**

The pregnancy-specific glycoprotein (*Psg*) genes encode proteins of unknown function, and are members of the carcinoembryonic antigen (*Cea*) gene family, which is a member of the immunoglobulin gene (*Ig*) superfamily. In rodents and primates, but not in artiodactyls (even-toed ungulates / hoofed mammals), there have been independent expansions of the *Psg *gene family, with all members expressed exclusively in placental trophoblast cells. For the mouse *Psg *genes, we sought to determine the genomic organisation of the locus, the expression profiles of the various family members, and the evolution of exon structure, to attempt to reconstruct the evolutionary history of this locus, and to determine whether expansion of the gene family has been driven by selection for increased gene dosage, or diversification of function.

**Results:**

We collated the mouse *Psg *gene sequences currently in the public genome and expressed-sequence tag (EST) databases and used systematic BLAST searches to generate complete sequences for all known mouse *Psg *genes. We identified a novel family member, *Psg31*, which is similar to *Psg30 *but, uniquely amongst mouse *Psg *genes, has a duplicated N1 domain. We also identified a novel splice variant of *Psg16 *(*bCEA*). We show that *Psg24 *and *Psg30 */ *Psg31 *have independently undergone expansion of N-domain number. By mapping BAC, YAC and cosmid clones we described two clusters of *Psg *genes, which we linked and oriented using fluorescent *in situ *hybridisation (FISH). Comparison of our *Psg *locus map with the public mouse genome database indicates good agreement in overall structure and further elucidates gene order. Expression levels of *Psg *genes in placentas of different developmental stages revealed dramatic differences in the developmental expression profile of individual family members.

**Conclusion:**

We have combined existing information, and provide new information concerning the evolution of mouse *Psg *exon organization, the mouse *Psg *genomic locus structure, and the expression patterns of individual *Psg *genes. This information will facilitate functional studies of this complex gene family.

## Background

In mammalian pregnancy the interaction between the maternal uterine tissues and foetal trophoblasts is regulated by a wide variety of cellular and endocrinological mechanisms. These mechanisms underpin trophoblastic invasion and remodelling of maternal tissues, placental angiogenesis, and the modulation of maternal immune responses. Central to these processes is the production by trophoblast of a variety of hormones that are found in abundance in the maternal bloodstream during pregnancy [1].

The pregnancy-specific glycoproteins (PSG) are the most abundant foetal proteins in the maternal bloodstream in late pregnancy [2]. They are synthesised in the syncytiotrophoblast of the human placenta and giant cells and spongiotrophoblast in the rodent placenta [3-5]. The PSG family of glycoproteins belongs to the carcinoembryonic antigen (CEA) family, which also includes the CEA-related adhesion molecules (CEACAMs). The CEA family is itself part of the immunoglobulin (Ig) superfamily [6]. The Ig domain structure of the human and rodent PSGs differs. Containing both V-like Ig domains (N), C2-like Ig domains (A and B) and relatively hydrophilic tails (C), domain arrangements in human PSGs are type I (N-A1-A2-B2-C), type IIa (N-A1-B2-C), type IIb (N-A2-B2-C), type III (N-B2-C) and type IV (A1-B2-C) [7]. In contrast, rodent PSGs are typically comprised of 3, and in a few cases of 5 or 7 N-domains followed by an A-domain [8]. In the primate / rodent ancestor, the initial duplication of the CEACAM / PSG primordial gene has been estimated to have occurred about 90 Myr ago [9], approximately at the time of human-rodent divergence. The most probable PSG ancestor in rodents and primates is a CEACAM15-like molecule based on the organisation of N and A domains. CEACAM15 is not classified as a PSG because comparisons of N and A domain sequence identity clearly delineate members of the CEACAM and PSG subfamilies (Roland Zebhauser, WZ, AM, TM, to be published elsewhere). It has been suggested that human and rodent PSG multigene families evolved independently via further gene duplication and exon shuffling events [10].

There are 11 members of the PSG family in humans that are encoded by genes clustered on chromosome 19q13.2 [11,12]. PSG proteins have a similar domain structure to the CEACAMs, but lack a membrane anchor and are therefore secreted. However, a few variants have been described that are retained within the cell. Conversely, a small number of human and mouse CEACAM variants lack a membrane anchor and are secreted. Membrane-anchored CEACAMs are widely expressed during embryonic development and in adult tissues, and are implicated in carcinogenesis, angiogenesis and regulation of immune functions [13,14]. In contrast, PSGs and some CEACAMs are expressed almost exclusively in trophoblasts of the haemochorial placenta of rodents and primates [4,5,15].

The biochemical properties and physiological functions of the PSGs remain to be fully elucidated, although functional experiments and clinical observations are beginning to provide some clues. Low PSG levels in the maternal circulation are associated with threatened abortions, intrauterine growth retardation and foetal hypoxia [16-19]. The importance of PSGs for the maintenance of pregnancy is also underlined by the observation that the application of anti-PSG antibodies or vaccination with PSG induces abortion in mice and monkeys, respectively, and reduces the fertility of non-pregnant monkeys [20,21]. The majority of PSG functional studies have focussed on determining whether PSGs are able to modulate the maternal immune system to prevent rejection of the allotypic foetus. Early studies with complex PSG mixtures isolated from placenta indicated an inhibitory effect on phytohaemagglutinin or allogeneically stimulated lymphocytes [22,23]. In further experiments it was shown that human monocytes secreted anti-inflammatory cytokines in response to PSG exposure. Moreover, recombinant mouse PSG18 was found to induce the production of interleukin (IL)-10 in the mouse macrophage cell line RAW 264.7 [24]. Human PSG1, PSG6 and PSG11 all induced secretion of IL-10, IL-6 and transforming growth factor (TGF)-β1 [25]. Whilst IL-10 and TGF-β1 are anti-inflammatory [26], IL-6 is usually considered to be a proinflammatory cytokine. However IL-6 does have some well-described anti-inflammatory properties [27]. Furthermore, IL-6 has been shown to indirectly promote trophoblast growth by upregulation of human chorionic gonadotropin (hCG) release by the trophoblast, and induction of granulocyte-macrophage-colony stimulating factor (GM-CSF) [28,29]. Further evidence implicating PSGs in immune modulation arises from PSG mediated suppression of T cells in purulent septic complications of abortion [30] and elevated circulating PSG levels are correlated with improved symptoms of rheumatoid arthritis [31]. PSG induction of alternative monocyte activation is of particular importance as it implies a PSG-mediated switching of the immune system from a predominantly T_H_1 response to a predominately T_H_2 response which is more compatible with a successful pregnancy [32].

The only PSG receptor identified to date is the integrin-associated CD9 receptor, which was found to bind the N1 domain of both PSG17 [33] and PSG19 (unpublished data). Additionally, the presence of the conserved tripeptide motif Arg-Gly-Asp (RGD) on a solvent-exposed loop in the N-terminal Ig domain in the majority of human and some lower-primate PSGs implicates a function that involves integrin-related receptors [34]. Thus it has been speculated that the RGD domain may enable some PSGs to disrupt cell-matrix interactions [35]. However, no rodent PSG isolated to date possesses an RGD domain. Evidence supporting the hypothesis that the RGD domain may be involved in receptor binding was provided by the discovery that a peptide containing the RGD motif, from human PSG9, bound to a receptor on the surface of a promonocytic cell line [36]. In common with integrin interactions, this was dependent on the presence of divalent cations and showed sensitivity to cytoskeletal signalling. However, the expected sizes of the receptor subunits differed from those of known integrins, therefore, the identity of the receptor remains elusive.

Much current work has focussed on human PSGs due to their possible relevance to disorders of pregnancy. However, the study of rodent PSGs is important because, the evident differences between primate and rodent PSG protein domain structures notwithstanding, there appears to be considerable conservation in terms of expression in trophoblast, independent gene family expansions in mammalian lineages with haemochorial placentation, and postulated immune functions during pregnancy. Moreover, the application of gene targeting and mutagenesis in the mouse is likely to be informative with respect to elucidating the cellular and physiological functions of PSGs. Such experiments will require an accurate genomic map of the mouse *Psg *locus, which we undertook to produce in the work described herein. It is also pertinent to ask whether the independent expansions of *PSG *gene families in different mammalian lineages reflect selection for increased gene dosage or for diversification of function mediated through different protein structures or developmental expression patterns. We therefore undertook to examine and correlate protein domain evolution and expression profiles of the various mouse *Psg *genes to attempt to address this question. Our results suggest that different family members have very different expression levels at different stages of development, which we consider may be supportive of the hypothesis that mouse *Psg *genes may have evolved divergent functions in mammalian pregnancy. However, mutagenesis of individual family members will be necessary to rigorously test this hypothesis.

## Results

### Identification of novel mouse *Psg *genes

For comparative studies of the human PSG family it is relatively easy to compare coding sequences (CDS) and peptide sequences because complete sequence information is available. However the data available for mouse PSGs is not complete, making such analyses difficult. Thus, we firstly collated the currently available public data, and we then attempted to identify sequences for PSGs that were not completely resolved in the databases. Full-length cDNA sequences of *Psg17*, *Psg18*, *Psg19*, *Psg21*, *Psg23*, *Psg28 *and *Psg30 *were identified via basic name searches of the RefSeq RNA database. Their identity was then verified by comparison to cDNA fragment sequences, which were obtained during the course of this work and deposited in GenBank [37], as misnaming of genes is commonplace in the databases. The cDNA sequence of *Psg22 *was then identified via BLAST analysis of the mouse RefSeq RNA database using the GenBank partial sequence referenced in Beauchemin *et al*. [37]. *Psg31 *was identified by BLAST analysis of the same database using the full-length *Psg30 *sequence and found to be the XM_355864.1 predicted transcript. However, there was a discrepancy between the predicted transcript and the sequences of EST clones CK032208 and CN694284. Comparison of these EST sequences with genomic contig NT_039395.2, using pairwise BLAST analysis, revealed that there had been a duplication of the N1 domain exon. We refer to the two N1 domains of *Psg31 *as N1 and N1* hereafter.

The gene and full-length cDNA coding sequences of the remaining mouse genes (*Psg20*, *Psg24*, *Psg25, Psg26*, *Psg27 *and *Psg29*) were deduced manually by systematic BLAST analysis of the mouse genome database as described in Methods. None of these predicted cDNAs were observed in the mouse EST database, although all except *Psg20 *were observed in the Trace Archive EST sequences. A novel splice variant of *Psg16 *was also found. BLAST analysis of the mouse High Throughput Genomic Sequences (HTGS) database identified contig AC148976.2, which appears to contain the whole *Psg16 *gene. An alternative exon 1 was discovered upstream of the previously described initiating exon by a pairwise BLAST comparison of this contig with full-length *Psg17 *coding sequence. The use of this alternative exon 1 produced a transcript that encodes a typical PSG polypeptide complete with a predicted secretory-peptide signal sequence and cleavage site. Multiple hits identified from subsequent BLAST analysis of the mouse EST and Trace Archives EST databases provided evidence that this novel splice variant was placentally expressed *in vivo*. In contrast, only one hit was obtained by identical analysis using the coding sequence of the brain-specific transcript described in Chen *et al*. [38]. This transcript (BC030357) was derived from a retinal cDNA library. The brain-expressed splice variant is generated from an alternative initiation site within exon 2 of the dominant placentally-expressed form of the gene. Alternative promoter usage would explain the brain and placenta-specific expression patterns of these variants of *Psg16*. Unlike the brain-specific variant, the placentally-expressed variant possesses a predicted secretory signal peptide at the N-terminus, like most other *Psg *gene family members.

The comparison of the brain derived *Psg16 *coding sequence with the genomic sequence (AC148976.2) also revealed differences in the encoding of the A-domain. The placental transcript is predicted to be encoded by 5 exons, as are the majority of mouse *Psg *mRNAs. However, a weak splice donor signal sequence within the fifth exon permits splicing to a strong splice acceptor sequence downstream of the sixth exon, as seen in the brain-expressed transcript. Trace Archive EST data reveals multiple hits to sequences from placental cDNA libraries using the 3' end of the placental *Psg16 *coding sequence as bait. This confirms the existence of our predicted transcript. Conversely, similar analysis using the brain-expressed variant yielded no hits. The sixth exon is present on a separate randomly ordered gene fragment within the AC148976.2 contig.

*Psg-ps1 *was previously considered to be a pseudogene, based on a point deletion at nucleotide position 30, downstream from the canonical *Psg *translational start site [8]. However, despite this frame shift, the open reading frame of this unusual *Psg *continues 105 bp upstream of the site of the mutation to an alternative ATG. Inspection of the sequence revealed a Kozak consensus, and BLAST analysis of the public EST and Trace Archive EST databases yielded many mRNA clones that contain this region in addition to downstream exons. Hence, this gene is clearly expressed, and we now propose to rename *Psg-ps1 *as *Psg32 *hereafter. We note that this mutation and amino terminal extension abolishes the canonical PSG secretory signal and peptide cleavage site. We therefore suggest that if *Psg32 *is indeed translated, the resulting protein is retained within the cytoplasm. To determine if the deletion observed in BALB/c mice was also present in other murine strains, we amplified and sequenced a 146 bp fragment by PCR using a set of primers specific for the 5'-untranslated region and the leader peptide of Psg32. The deletion observed in the *Psg32 *cDNA is also present in the genomic DNA of A/J, C57BL6/J, YBR/Ei, and SWR/J inbred mouse strains (data not shown).

The nomenclature (past and current) and accession numbers of nucleotide sequences of all the murine PSGs are documented in Table 1. The genome sequence and predicted CDS and translation products for *Psg16*, *Psg20*, *Psg26 *and *Psg31 *are listed in Additional file 1. The complete CDS data for all known mouse *Psgs *(except the brain-specific splice variant of *Psg16*) are listed in Additional file 2. The complete protein primary sequences for all known mouse Psgs (except the brain-specific splice variant of *Psg16*) are listed in Additional file 3.

**Table 1:** Summary of mouse PSG nomenclature and sequence accession numbers

**Current Name**	**Previous Names**	**Accession Number^a^**	**Comment^b^**
Psg16	bCEA	AC148976.2 (RC 40000–60000)	predicted CDS: join (1878–1941, 4115–4462, 7291–7650, 8750–9109, 11758–12041); *bCEA is a splice variant of Psg16*
Psg17	Cea2, mmCGM5	NM_007677	
Psg18	Cea3, mmCGM6	NM_011963	
Psg19	Cea4	NM_011964	
Psg20	Cea7	AC079497.1 (113793–127892)	predicted CDS: join (1770–1836, 2989–3345, 4997–5356, 6587–6937, 13114–13397)
Psg21	Cea8	NM_027403	
Psg22	Cea9	NM_001004152.1	
Psg23	Cea11	NM_020261	
Psg24	Cea12	AC079526 (115000–131000)	predicted CDS: join (1648–1696, 2771–3130, 5965–6324, 9351–9710, 10943–11302, 12844–13191, 14196–14479)
Psg25	Cea13	NW_000292.1 (RC 890000–910000)	predicted CDS: join (4905–4968, 7121–7480, 10406–10765, 11988–12347, 15508–15791)
Psg26	Cea14	join (CAAA01217140.1 {RC 1–6315}, CAAA01213459.1 {557–4715}, CAAA01175422.1 {155–2891})	predicted CDS: join (2148–2211, 3292–3651, 5836–6195, 7507–7866, 10823–11106)
Psg27	Cea15	AC087156.1 (RC 139366–153050)	predicted CDS: join (240–303, 2037–2393, 5271–5630, 6669–7028, 10039–10322)
Psg28	Cea16	NM_054063	
Psg29	Cea17	AC079526 (183285–194009)	predicted CDS: join (1459–1522, 2658–3005, 6275–6634, 8128–8487, 9428–9700)
Psg30		XM_145406	GNOMON prediction in NCBI
Psg31		AC134475.3 (10000–70000)	predicted CDS: join (3923–3986, 5262–5621, 19366–19725, 34382–34741, 36822–37172, 40760–41119, 42413–42763, 47310–47669, 49090–49443, 50473–50756)
Psg32	Psg-ps1	XR_000250	GNOMON prediction in NCBI

^a ^Where nucleotide start and end positions are shown in parenthesis after accession numbers, they refer to the start and end positions of the genomic sequence excerpt (encompassing the PSG exons) that is included in Additional file 1. RC indicates that the sequence in Additional file 1 is the reverse complement. ^b ^Where we have predicted the full CDS of a PSG (based on common structure and splice sites), the numbers shown refer to exon start and end positions within the excerpted sequence included in Additional file 1.

### Domain structure of mouse PSG proteins

**Figure 1:**
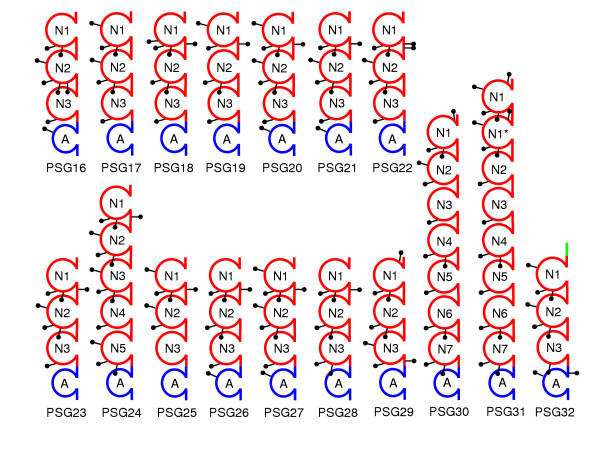
**Domain organization of mouse PSGs**. Mouse PSGs are composed of 3 – 8 IgV-like N domains and one IgC-like A domain. The relative position of potential N-glycosylation sites (consensus amino acid sequence: asparagine-X-threonine / serine; X any amino acid except proline) were identified using the NetNGlyc 1.0 Server online software  and indicated by lollipops. Although PSG32 is probably not routed through the endoplasmic reticulum, the putative N-glycosylation sites are shown for comparison. Of the two PSG16 splice variants, only the variant expressed in the placenta is shown.

**Figure 2:**
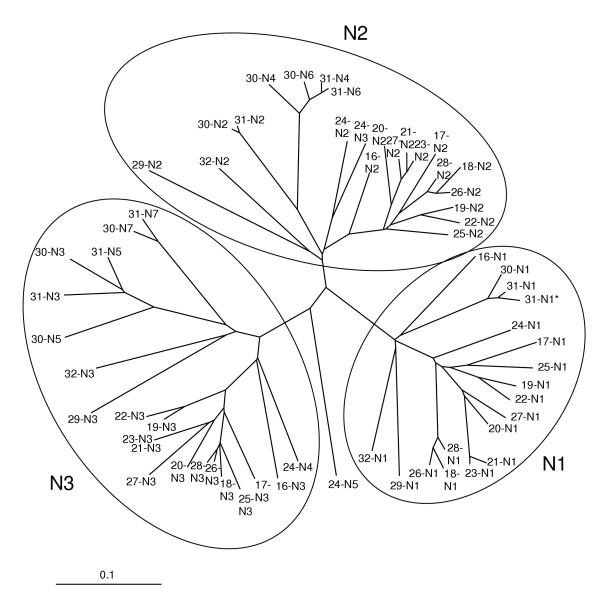
**Evolutionary relationships between mouse PSG IgV-like domains**. An unrooted evolutionary tree based on ClustalX amino acid sequence alignments showing the relationships between all mouse PSG N-domains. The three main groups N1, N2 and N3 have been ringed for clarity. The scale bar represents 0.1 amino acid substitutions per site.

A schematic representation of the mouse Psg domain structures is shown in Fig. 1. Of the seventeen mouse *Psgs*, thirteen encode a common structure of three Ig variable (IgV)-like domains (N-domains) and a single Ig constant (IgC)-like domain (A-domain). *Psg24*, *Psg30 *and *Psg31 *have an expanded structure created by the duplication of IgV-like domains. An unrooted phylogenetic tree indicates three main branches of IgV-like domain evolution (Fig. 2). There is a group consisting of N1 domains, a group of N2 domains and N2-derived domains, and a group of N3 domains and N3-derived domains. Therefore, in agreement with the most common structure observed in Fig. 1, the ancestral mouse Psg would be expected to have had an N1-N2-N3-A arrangement of domains. The expansion of *Psg24*, *Psg30 *and *Psg31 *has occurred mostly through duplications of the N2 and N3 IgV-like domains, with the exception of *Psg24 *N5 and *Psg31 *N1 domains.

**Figure 3:**
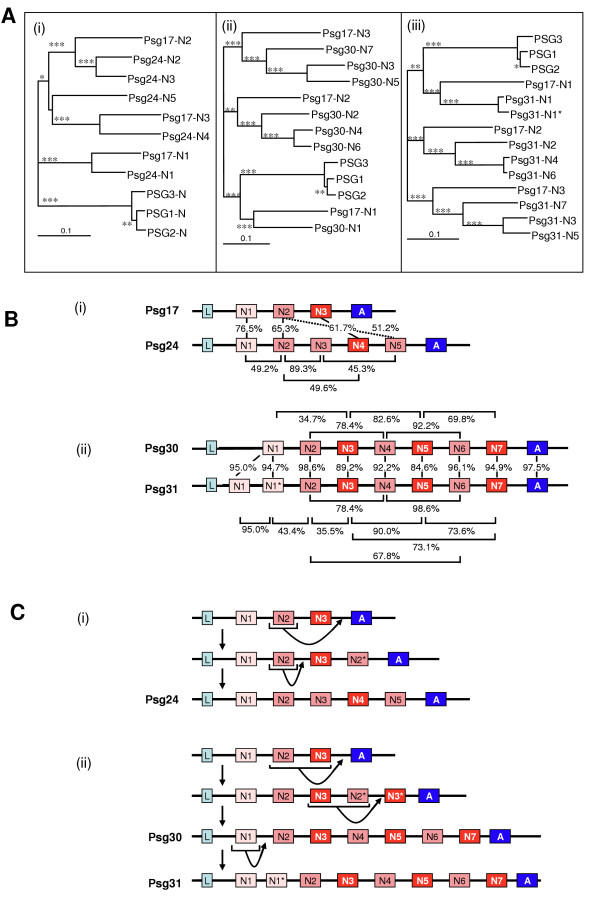
**Domain expansion of *Psg24*, *Psg30 *and *Psg31***. A. NJ-trees based on ClustalX amino acid sequence alignments showing: (i) the evolution of PSG24 IgV-like domains compared to those of PSG17; (ii) the evolution of PSG30 IgV-like domains compared to those of PSG17; (iii) the evolution of PSG31 IgV-like domains compared to those of PSG17. The trees were rooted using an outgroup consisting of the N-domain amino acid sequences of human PSG1, PSG2 and PSG3. Alignments were bootstrapped 1000 times yielding node values which are represented as follows < 50%: no mark; 50–74%: marked *; 75–94%: marked **; ≥ 95%: marked ***. The scale bar represents 0.1 amino acid substitutions per site. B. The arrangement of domains represented by boxes shaded: cyan for leader (L) peptides; light pink for the N1-domains; dark pink for N2 and N2-like domains; red for N3 and N3-like domains; blue for A-domains. (i) Comparison of *Psg17 *and *Psg24 *exon arrangement including identities of amino acid sequence alignments. (ii) Comparison of *Psg30 *and *Psg31 *exon arrangements including identities of amino acid sequence alignments. C. Predicted model of IgV-like domain expansion by exon duplications in (i) *Psg24 *and (ii) *Psg30 *and *Psg31*.

In order to characterise the evolution of the mouse *Psg*s with expanded domain numbers, Neighbor-Joining (NJ) trees with bootstrap values of 1000 were prepared (Fig. 3A) and ClustalW amino acid sequence alignments (Fig. 3B) were studied to identify the origin of the novel IgV-like domains in these three exceptional *Psg*s. From examination of the data in Fig. 3A(i) it was not apparent from which progenitor domain the *Psg24 *N5 domain evolved due to lack of confidence in the branch on which it lies. However, using the alignment identities in Fig. 3B(i) it can be seen that, although generally poorly conserved, the best match of 51.2% was obtained by alignment with the N2 domain. Therefore, our evolutionary model assumes that the *Psg24 *N5 domain arose from an early duplication of the N2 domain. Also, based on agreement of the data in Fig. 3A(i) and 3B(i), the N2 domain duplicated again more recently to yield the N3 domain. This latter duplication explains why the *Psg24 *N4 domain is N3-like. The order of these events is shown schematically in Fig. 3C(i).

Using a similar analysis we propose a model for the expansion of domains within *Psg30 *and *Psg31 *(Fig. 3C(ii)). We suggest that the N4 and N6 domains of *Psg30 *and *Psg31 *are derived from a progenitor N2 domain. Similarly, the N5 and N7 domains are derived from a progenitor N3 domain. Expansion is predicted to have occurred in 2 or 3 separate events in a common ancestor of *Psg30 *and *Psg31*. In the first instance the progenitor N3-like and N2-like domains were duplicated, either at different points in evolution or at the same time. The final step was a duplication of both of these daughter domains to create *Psg30 *and the precursor of *Psg31*. The precursor of *Psg31 *then underwent another duplication, this time of the N1 domain.

### Expression of *Psg *genes in mouse placenta at different developmental stages

**Figure 4:**
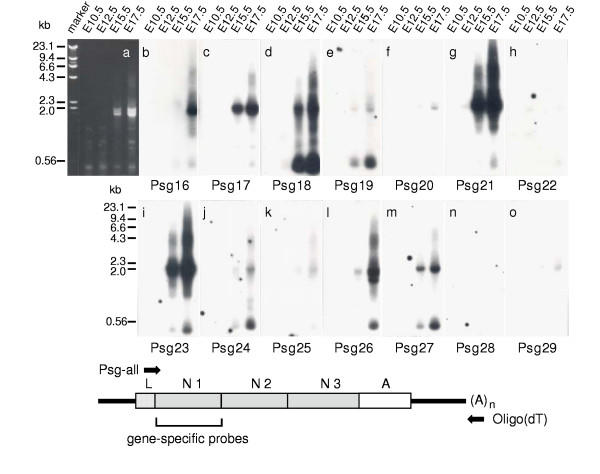
**Expression of *Psg *mRNAs during placental development**. Total RNA (1 μg) from day 10.5, 12.5, 15.5 and 17.5 BALB/c placentae was reverse transcribed using an oligo (dT) oligonucleotide (reverse PCR primer). After addition of the degenerate Psg-all oligonucleotide (forward PCR primer), which anneals to the cDNA of all known members of the mouse *Psg *family, *Psg *cDNAs were amplified by PCR (see schematic diagram depicting generalised mouse *Psg *cDNA amplification). Aliquots were size-separated by agarose gel electrophoresis. a, PCR products were visualised by ethidium bromide staining. b-o, the amplification products were blotted onto nylon membranes and individual blots were hybridised with single gene-specific 32P-labelled oligonucleotides from the N1 domain regions (Table 2). The location of the primers used for amplification of the *Psg *cDNAs and the region from which the sequences of the gene-specific oligonucleotides were derived are shown together with a schematic representation of mouse *Psg *mRNA. The 5'- and 3'-untranslated regions are shown as bold lines. L, leader; N1-N3, IgV-like domains; A, IgC-like domain.

On the basis that all mouse *Psg *genes originated from a common ancestor, and expanded into a multigene family by duplication and subsequent divergence, the question as to whether the expression patterns have also diversified is relevant to determining the selective forces underlying *Psg *gene family expansion. As *Psg *genes are expressed predominantly in the placenta, cDNA was prepared from total RNA extracted from mouse placenta at four stages of development between E10.5 and E17.5. *Psg *cDNA sequences were then amplified with PCR primers designed to amplify *Psg16 *– *Psg29 *inclusive. Size fractionation of PCR products on an ethidium bromide-stained agarose gel, indicates that mouse *Psg *genes are predominantly expressed from around E15.5, increasing in expression through to at least E17.5 (Fig. 4). However, after blotting the products onto nylon membranes and hybridising radiolabelled oligonucleotide probes specific for individual *Psg *genes (Table 2), we observed significant differences in expression profiles of different genes during development. This method is probably semi-quantitative at best but does give some indication of relative expression levels. We observed that *Psg16 *and *Psg26 *are weakly expressed at E15.5 but strongly expressed at E17.5. In contrast, *Psg17*, *Psg18*, *Psg21 *and *Psg23 *are expressed strongly at E15.5, further increasing by E17.5. *Psg27 *shows a similar expression pattern to these four *Psgs*, but at a relatively low level. Very weak expression was observed on E17.5 for *Psg19*, *Psg20*, *Psg24*, *Psg25 *and *Psg29*, whereas *Psg22 *and *Psg28 *were undetectable. *Psg30*, *Psg31 *and *Psg32 *domain structures had not been finalised and therefore their expression was not analysed in this experiment.

**Table 2:** Oligonucleotides used in this study

**Oligo**	**Sequence**	**Position**	**Comment**
Psg17A5'	5'-CTTGCCACACAGCCCGTCAT-3'	Psg17 A domain	
Psg17A3'	5'-TCATCACAGCCAGGATGACT-3'	Psg17 A domain	
mPsg-5'	5'-AWCCTSYTGSYTCCTGC-3'^a^	N1 domain	binds to several mouse Psg cDNA sequences
mPsg-3'	5'-TGMARGWAYAKGGATGT-3'^a^	N1 domain	binds to several mouse Psg cDNA sequences
PsgN1-F	5'-GAAGATCTAGCCTCCMTYTTDDCCT-3'^a ^*Bgl *II	intron 1/N1 exon	for the amplification of all known *Psg *N1 exons (except *Psg32*)
PsgN1-R	5'-CCATCGATTACTTACWGTWSACVTRVA-3'^a ^*Cla*I	N1 exon/intron 2	for the amplification of all known *Psg *N1 exons (except *Psg32*)
Psg32N1-F	5'-GAAGATCTAGCTTTTCTTTTAACCTC-3' *Bgl *II	N1 domain	
Psg32-exon1	5'-GAGGTGTCCTTGGTGCTTCTC-3'	exon 1	Psg32-specific
oligo (dT)	5'-TTCTAGAATTCAGCGGCCGC(T)_30 _VN-3'^a^	poly(A) tail	
Psg-all	5'-CCTCCMTYTTDDCCTRCTGS-3'^a^	N1 domain	binds to all known Psg cDNA sequences except Psg32
bCEAN/2	5'-GCAAATGTACAGTGGTAG-3'	N1 domain	Psg16-specific
Psg17N	5'-GTGGAATTCTTACCTCCC-3'	N1 domain	Psg17-specific
Psg18N	5'-GGCTGTACTACTATAGTG-3'	N1 domain	Psg18-specific
BK07	5'-AAAGTGCCACCCGGGAA-3'	N1 domain	Psg19-specific
Psg20N	5'-TGCCAAGGTCACTATCCA-3'	N1 domain	Psg20-specific
Psg21N	5'-GCTCTGCATTTTCTGGAC-3'	N1 domain	Psg21-specific
35N	5'-GTCTGGTATAGAGGGGTG-3'	N1 domain	Psg22-specific
53N	5'-GCTGTGTATTTACTGGAC-3'	N1 domain	Psg23-specific
9.3N1	5'-ATAGCAGAGGTGTGACG-3'	N1 domain	Psg24-specific
11.2N1	5'-ATCTTCTAGGCCTTGCC-3'	N1 domain	Psg25-specific
189N	5'-CATTCGCTGTACTATAGTG-3'	N1 domain	Psg26-specific
214N	5'-CGAGTCACCATCCATTCA-3'	N1 domain	Psg27-specific
2128N	5'-GCACTATAGTTTAACAGCG-3'	N1 domain	Psg28-specific
9140N	5'-TGCAGTGGTGTCTGACTT-3'	N1 domain	Psg29-specific
Psg-ps1N	5'-TTAGTGCCACCACAAGTG-3'	N1 domain	Psg32-specific

^a ^Standard IUB/IUPAC nucleic acid codes codes have been used to indicate degeneracy where: R = G/A; Y = T/C; K = G/T; M = A/C; S = G/C; W = A/T; B = G/T/C; D = G/A/T; H = A/C/T; V = G/C/A; N = A/C/G/T.

**Figure 5:**
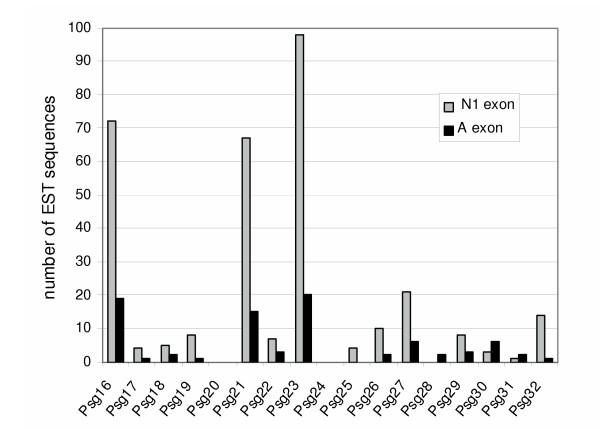
**Virtual Northern analysis of the mouse *Psg *genes**. The nucleotide sequences of the *Psg *exons encoding the N1 or the A domains were used in NCBI-BLAST searches of the GenBank mouse EST database (March 16, 2004) for the presence of *Psg *transcripts (virtual Northern analysis). A hit was registered when a 100% match for a sequence > 150 bp was observed. Obvious mismatches such as unidentified nucleotides (N) or single nucleotide insertions or deletions (especially at the end of a sequence run) were ignored.

To supplement the PCR-based *Psg *expression studies, we performed 'virtual northern' analysis *in silico *by screening the public EST database for sequences matching *Psg *N1 or A-domains and counting the numbers of matches (Fig. 5). There was generally good concordance of the virtual data with the RT-PCR data; notably, *Psg21 *and *Psg23 *are highly represented in both datasets. However, disagreements were also evident e.g. *Psg16 *expression was low in the RT-PCR data, but high in the virtual data. A random sample of twenty of the large number of *Psg16 *EST sequences in the database indicated that all were of placental origin, ruling out contamination with brain-derived sequences as an explanation for the disparity between RT-PCR and virtual analysis. There was also generally good agreement with the results from screening the EST database with N1 and A domain sequences, although the numbers of A-domain hits were 4–5 fold lower than the N1-domain hits. The only exception to this observation was that *Psg30 *and *Psg31 *sequences were identified in 2-fold greater abundance when screened with the A domain compared with the N1 domain. Despite some discrepancies, therefore, the combined RT-PCR and virtual Northern data demonstrate that developmental onset of expression, and maximum expression levels, vary considerably within the *Psg *family.

### Mouse *Psg *locus genomic organisation

**Figure 6:**
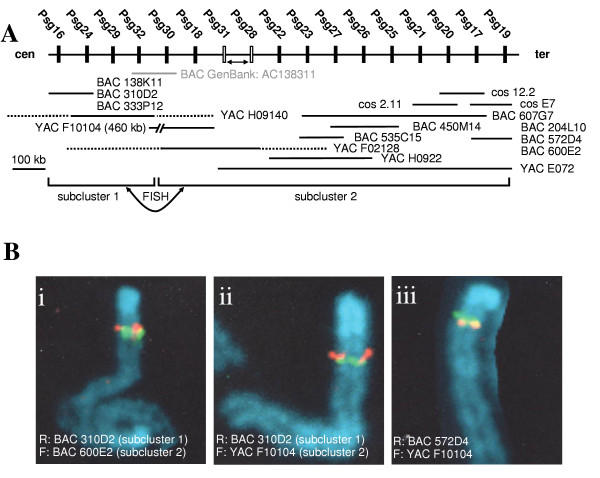
**Physical map of mouse *Psg *gene locus**. A. The order of the *Psg *genes was inferred from the presence of the various genes on overlapping cosmid, BAC and YAC clones. The position of *Psgs *represented by filled boxes is unequivocal, whereas the position of those represented by open boxes is ambiguous. Arrows between pairs of genes indicate that their order remains unresolved. The distances between individual genes are not shown to scale. Chimeric YACs mapping to separate chromosomes are indicated by stippled and solid lines. The solid lines correspond to chromosome 7 regions containing the *Psg *genes indicated above. The locations of the non-chromosome 7 regions are not known. Only the sizes of non-chimeric YACs have been determined and are shown (size bar corresponds to 100 kb). The centromere (cen) / telomere (ter) order and the relative orientation of the two *Psg *gene subclusters were resolved by FISH mapping. B. Two-colour FISH prophase mapping of relative orientation of the two *Psg *gene subclusters using mouse m5S cells and C57BL/6CrSlc mouse lymphocytes. (i) FISH pattern representative of 38 experiments where BAC 310D2 in subcluster 1, labelled with rhodamine (R), is centromeric to BAC 600E2 from subcluster 2, labelled with fluorosceine (F). (ii) FISH pattern representative of 38 experiments where BAC 310D2 in subcluster 1, labelled with rhodamine, is centromeric to YAC F10104 from subcluster 2, labelled with fluorosceine. (iii) Orientation of subcluster 2 determined by relative positions of BAC 572D4, labelled with rhodamine, which is telomeric to YAC F10104, labelled with fluorosceine.

The published mouse *Psg *gene locus is contained on contig NT_039395. However, the complement of *Psg *genes is incomplete and the majority of gene sequences within the contig are unordered. We therefore decided to determine the organisation of *Psg *genes within the locus by screening BAC, YAC and cosmid clones using hybridisation with gene-specific oligonucleotide probes. We defined two separate contigs (subclusters) within which the order of *Psg *genes was determined to the fullest extent possible. The orientation of the two subclusters with respect to each other and the chromosome 7 centromere was determined by fluorescent in situ hybridisation (FISH) analysis. These data are summarised in Fig. 6. All of the known mouse *Psg *genes are located within cytobands A1 and A2 on proximal chromosome 7 and are interspersed with other genes, particularly *Ceacams*, as determined by comparison with the published mouse genomic sequence on contig NT_039395. We did not observe any obvious correlation between the relative positions of the *Psg *genes at the locus and their domain arrangements or expression patterns.

There is a discrepancy with respect to the distance between the two subclusters. The currently poorly resolved data covering this region in the the Ensembl assembly implies the presence of a gap between *Psg29 *and *Psg32*. However, we determined that the subclusters are fused between *Psg32 *and *Psg30/Psg18*. YAC F10104 (which is non-chimeric) is about 460 kb long and contains only two *Psg *genes which indicates the presence of a non-*Psg *genomic region. We estimate the gap between the subclusters to be approximately 400 kb based on the size of cosmids containing two *Psg *genes (ca. 40 kb).

## Discussion

The human *PSG *genomic data in the public databases are relatively complete. For each *PSG *gene, there are annotated RefSeq resources comprising information on genomic structure, transcripts and translation products. The nomenclature is also standardised [37]. Further, there are accurate chromosome 19 locus assignments allowing complete visualisation of the *PSG *locus and surrounding genes. In contrast, a substantial quantity of mouse *Psg *genomic data in the public domain is fragmented, incomplete and somewhat unreliable. We sought to collate the existing genomic data, to present novel data to fill in gaps, and to provide a coherent resource of mouse *Psg *genomic data.

To determine whether the existing set of mouse *Psg *genes was complete we performed systematic BLAST searches of a variety of public DNA sequence databases. This analysis revealed the existence of a novel expressed *Psg *gene, which we name *Psg31 *in line with the accepted nomenclature convention [37]. *Psg31 *apparently evolved from a duplication of the whole of the *Psg30 *gene followed by a subsequent internal duplication of the N1 domain. We were also able to predict the complete coding sequences of four *Psg *genes for which previously only partial fragments were described. The gene, CDS and protein sequences of these predictions, coupled with a complete reference of all known mouse *Psg *CDS and primary protein sequences are provided in three attached Additional Files.

Using the full CDS information obtained for the complete set of mouse *Psg *gene sequences, domain structures for all family members were predicted. All of the PSG proteins possess previously described arrangements of Ig-like domains. Except for two members, discussed below, all are predicted to encode N-terminal secretory signal sequences. Our predicted novel splice variant of *Psg16 *has a complete N1 domain and secretory signal peptide sequence. Trace Archive EST database BLAST analysis confirmed that this variant is expressed in the placenta. In contrast, the brain-expressed variant [38] has only a partial N1 domain and no secretory signal peptide. The previously described *Psg-ps1 *pseudogene [8] was found to be expressed in the placenta using Trace Archive EST database BLAST analysis and possesses an excellent Kozak sequence at the predicted translational initiation site. This evidence therefore indicates that this gene, which we rename *Psg32*, is not a pseudogene but a *bona fide *expressed *Psg *gene family member. The *Psg32 *transcript may encode a protein that is retained within the cytoplasm. We note that a precedent in the human exists in the form of a non-secreted splice variant of *PSG11 *[7].

*Psg31 *has the unusual N1-N1*-N2-N3-N4-N5-N6-N7-A domain structure. This newly characterised *Psg *gene has evolved from a duplication of the entire *Psg30 *gene followed by an internal duplication of the N1 domain. There may be functional significance associated with the N1 domain duplication. The complex nature of *Psg *gene evolution, including putative gene conversion and recombination events between family members [34], makes it difficult to analyse their evolution. Despite this, the data generated from ClustalX alignments and NJ trees enabled us to generate trees that allow prediction of the order of events of domain duplications in *Psg24*, *Psg30 *and *Psg31*. We note that the apparent route to domain number expansion differed between *Psg24*, and *Psg30 *and *Psg31*. The extra N-domains of *Psg24 *are derived from two duplications of the N2 domain. However, for *Psg30 *and *Psg31*, independent duplications of each of the N2 and N3 domains were probably followed by a secondary duplication of the daughter domains, possibly as a single event.

Gene expression data from RT-PCR of placental RNA and EST database analysis revealed considerable differences in the expression levels of different *Psg *genes. In this analysis *Psg21 *and *Psg23 *were the most abundant, consistent with a previous report of abundant *Psg23 *expression [39]. Whilst there was generally good agreement between the two methods of expression analysis we cannot determine, based on current data, whether *Psg *gene expression differences reflect selection for divergent functions, or increased gene dosage for enhancement of an existing function, because expression levels were uniformly low for many family members, and there was a general trend of increased expression during gestation. *Psg *transcripts are found from day 6.5 of embryonic development onward in primary trophoblast giant cells, later (from day 10.5) in spongiotrophoblast cells and, to a lesser extent, in a cell population in the deciduas basalis at day 14.5 [40]. At present, it is unclear whether the various *Psg *genes exhibit different cellular expression patterns, which might indicate divergent functions of the various PSGs. There are interesting parallels between the expansion of the *Psg *gene family and similar expansions of other placentally-expressed gene families such as the prolactin and growth hormone families [1], and the aspartic and cysteine proteases [41]. Such duplications may be a manifestation of parent-offspring conflict or inter-sibling rivalry over maternal investment [42].

Having collated all known mouse *Psg *gene protein coding sequences and protein domain structures, the mouse *Psg *genomic locus on chromosome 7 remained to be determined to complete a comprehensive resource for the analysis of *Psg *function. The NCBI build 32 composite mouse assembly data revealed that only four *Psg*s had been mapped. Other *Psg*s on contig NT_039395 are currently unordered. We therefore screened cosmid, YAC and BAC libraries, and orientated *Psg*-containing clones to identify, where possible, the order of *Psg *genes within the locus. We were not able to resolve all ambiguities in gene order on our map; however, where public database information is available, our data are in good agreement. We found no clear relationship between gene location and gene expression level suggesting that, within the *Psg *locus, each *Psg *gene is autonomously regulated.

## Conclusions

The evolution and physiological functions of the relatively understudied mouse *Psg *gene family are poorly understood. This is a feature shared with other placentally-expressed, multigene families such as the prolactin and growth hormone genes [1]. In order to provide a comprehensive resource to facilitate functional studies of mouse *Psg *genes, including the generation of mouse mutants with modified *Psg *gene expression profiles, we have collated the entire set of mouse *Psg *genes, their predicted encoded proteins, and their evolutionary histories. The complete CDS data will enable the cloning, over-expression, and gene targeting of individual or multiple mouse *Psg *genes. This will facilitate the elucidation of their function and, by extrapolation, their human homologues, which may be involved in diseases of pregnancy.

## Methods

### Isolation of cosmid, YAC and BAC clones

Cosmid libraries in pWE15 which were made from liver DNA of BALB/c and C57BL/6 mice were obtained from Dr. Edwin N. Geissler, Boston, MA, USA, and Stratagene (Heidelberg, Germany), respectively. They were screened for the presence of *Psg *gene-containing cosmids using a ^32^P-labelled, full-length *Psg *cDNA (2.1 kb *Kpn*I/*Xba*I fragment of pCea2b [8]) as a probe. The final wash was in 4x NaCl/Cit (1x NaCl/Cit is 0.15 M NaCl, 0.015 M sodium citrate pH 7.0), 0.1% SDS at 65°C. *Psg *gene-containing YAC clones were identified by hybridisation of DNA from YAC clones which were spotted at high density onto nylon membranes [43] with the same *Psg *cDNA probe under medium stringency conditions with a final wash in 4x SSPE (1x SSPE is 180 mM NaCl, 10 mM sodium phosphate pH7.4, 1 mM EDTA), 0.1% sodium dodecyl sulfate (SDS) at 65°C. The membranes with arrayed YAC clone DNAs were kindly provided by Dr. H. Lehrach, Max-Planck-Institut für Molekulare Genetik, Berlin. Two libraries were screened, 902 and 903, both in the vector pYAC4 [44] composed of 9216 clones each, containing spleen DNA of C3H and C57BL/6 mice, respectively. Filters with arrayed BAC clones containing genomic DNA from the embryonic stem cell line CJ7 (129/Sv strain) (CloneRanger™ BAC Human CTC, Invitrogen, Karlsruhe, Germany) were screened by hybridisation with a ^32^P-labelled probe consisting of the N1 domain exon sequences of 14 mouse *Psg *genes (*Psg17*–*Psg29 *and *Psg32*). The N1 domain exons were amplified individually by PCR using the degenerate primer pair PsgN1-F/PsgN1-R or Psg32N1-F/PsgN1-R (4 mM each) for Psg32 (Table 2) and cosmid clones (10 ng) with individual *Psg *genes as template in the presence of 1 U Taq polymerase and 4 mM MgCl_2 _in a total volume of 30 ml (annealing: 50°C, 30 s). N1 exons of *Psg28 *and *Psg29 *were released by digestion with *Sal*I and *Kpn*I from pUC18 (see below).

### Southern blot analysis of cosmid and YAC DNAs

DNA from YAC clones was isolated by CsCl equilibrium density gradient centrifugation in the presence of ethidium bromide, essentially as described [45], except that spheroblast formation was achieved by incubation for 90 min with 0.17 mg/ml lyticase (approx. 6,000 U/mg) from *Arthrobacter luteus *(Sigma, Deisenhofen, Germany). Two mg of cosmid or 0.5 mg of YAC DNA were digested with restriction endonucleases, size fractionated by electrophoresis on 1% agarose gels and blotted onto positively charged nylon membranes. To identify N1 and A domain exon-containing DNA fragments, the digested DNAs on the membranes were hybridised with ^32^P-labelled N1 (cosmid DNA blots only) and A domain probes from *Psg17 *and washed under medium stringency conditions (4x SSPE, 0.1% SDS, 65°C). The *Psg17 *N1 and A domain cDNA fragments used as probes were obtained by PCR (denaturation: 94°C, 15 s; extension: 72°C, 3 min; 30 cycles) using the mPsg-5'/mPsg-3' (annealing: 50°C; 30s) and Psg17A5'/Psg17A3' (annealing: 60°C, 30 s), primer pairs respectively, and the *Psg17 *cDNA clone pCea2b as template (Table 2; [8]).

### Identification of new *Psg *genes from YAC clones

N1 exons from unknown *Psg *genes were amplified by PCR (annealing: 52°C, 30 s; 30 cycles) in a total volume of 100 μl using 200 ng of YAC clone DNA as template, 1 U *Taq *polymerase, 3 mM MgCl_2 _and 4 mM each of PsgN1-F and PsgN1-R degenerate oligonucleotides (Table 2) which bind to the N1, but not N2 and N3 exons of all known mouse *Psg *genes (except *Psg32*). The product was purified by electrophoresis on a 1.8% agarose gel and subcloned into pUC18 after blunt-ending (SureClone ligation kit: Pharmacia, Freiburg, Germany). The N1 exons from two of the 10 newly identified *Psg *genes (*Psg28, Psg29*) were analysed by sequencing recombinant plasmids which did not hybridise with oligonucleotide probes specific for known *Psg *genes (Table 2).

### Mapping of the *Psg *locus

The presence of the different *Psg *genes within YAC, BAC and cosmid clones was first determined by PCR followed by hybridisation with oligonucleotides specific for individual *Psg *genes. DNA from *Psg*-containing YAC (100 ng) and cosmid clones (10 ng) were used to amplify the N1 domain exons of all known *Psg *genes in a total volume of 60 μl as described above. The N1 exon of *Psg32 *was amplified in a separate reaction using Psg32N1-F and PsgN1-R (Table 2) as primers under the same conditions used for the amplification of the other N1 exons. For the analysis of BAC clones, PCR was performed directly from the BAC-containing bacterial clones according to the supplier's protocol. Aliquots (3.5 μl) from the various PCR reactions were alkali-denatured, dot-blotted onto nitrocellulose and hybridised with individual ^32^P-labelled (final concentration: 0.3–1.2 × 10^6 ^dpm/ml), gene-specific oligonucleotides (Table 2) in 0.5 M sodium phosphate pH 7.2, 7% SDS, 1 mM EDTA over night at 40°C. The filters were washed twice for 20 min each in 2x SSPE at room temperature, followed by two washes in 6x SSPE, 0.1% SDS at a temperature 4°C below the calculated melting temperature of the hybrids [46]. Oligonucleotides containing at least 3 mismatches in comparison with the corresponding sequences of all known *Psg *and *Cea *subgroup members were designed using the computer program *Primer *[47]. The only exception is the *Psg19*-specific oligonucleotide which exhibits only 2 mismatches to the *Psg22 *sequence. However, the stringency of the post-hybridisation washes only allowed binding of oligonucleotides with a maximum of one mismatch. The specificity of the oligonucleotides and the hybridisation conditions was demonstrated on cosmid DNAs containing individual *Psg *genes. The identity of the *Psg *genes was verified by sequencing. No cross-hybridisation with other *Psg *genes was observed. The size of the YACs was determined by pulsed field gel electrophoresis followed by Southern blot hybridisation with the *Psg17 *cDNA clone pCea2b (see above) essentially as described previously [48].

### Fluorescence in situ hybridisation (FISH) analyses

The chromosomal location and chimerism of YAC clones were determined by FISH analyses, using B1-PCR of YAC DNA for probe preparation essentially as described [49]. Orientation and order relative to the chromosome 7 centromere and to each other of the two *Psg *gene subclusters was defined by FISH analysis using probes described in Fig. 6. FISH was performed essentially as described [50] on m5S cells [51] and concanavalin A-stimulated lymphocytes [52] from the C57BL/6CrS1c mouse strain.

### RNA isolation, RT-PCR and specific detection of *Psg *cDNAs

BALB/c mice were mated overnight, and the next day plugged females were designated as day 0.5 of gestation. Pregnant females were killed by cervical dislocation and placentae were dissected free of maternal tissue, immediately frozen in liquid nitrogen and stored at -70°C. Total RNA was extracted by the acid phenol method [53]. The expression of individual *Psg *genes was studied by RT-PCR followed by hybridisation of the products with gene-specific oligonucleotides. Total RNA (1 μg) from placentae of different gestational stages was reverse transcribed in a total volume of 10 μl by avian myoblastosis virus (AMV) reverse transcriptase (Promega, Mannheim, Germany) in the presence of 6 U/μl RNasin (Promega) using a degenerate oligo (dT)_30 _oligonucleotide (1 μM) as primer (Table 2). The reaction mix was adjusted to 1x *Taq *buffer (20 mM Tris-Cl, 16 mM (NH_4_)_2_SO_4_, pH 8.6), 3 mM MgCl_2 _and 0.4 mM dNTPs in a total volume of 100 μl. Amplification of all known *Psg *cDNAs (except for the cDNA of *Psg32 *(*Cea6*), which at the time of the experiment was presumed to be a pseudogene [8]) was achieved by PCR (denaturation: 94°C, 15 s; annealing: 58°C, 30 s; extension: 72°C, 3 min; 30 cycles) using *Taq *polymerase after addition of 400 pmoles of Psg-all (Table 2) and 50 pmoles of the oligo (dT) oligonucleotide as 5'- and 3'-primer, respectively. Ten μl aliquots each were size fractionated by electrophoresis on a 1% agarose gel, blotted onto a positively charged nylon membrane (Roche Diagnostics, Mannheim, Germany) and hybridised with individual ^32^P-labelled, gene-specific oligonucleotides (Table 2) as described above.

### DNA sequence determination

Nucleotide sequences were determined on both strands with flanking universal and internal oligonucleotides as primers using a T7 polymerase sequencing kit (Pharmacia) or a Taq Dye Deoxyterminator cycle sequencing kit (PE Applied Biosystems, Foster City, CA, USA).

### Assessment of availability of full-length mouse *Psg *sequences in the public databases

All bioinformatics searches described below used the online software and databases available at the NCBI . Where fully annotated, *Psg *cDNA sequences were identified by name searches of the RefSeq RNA database. Attempts were then made to identify remaining known *Psg *cDNA sequences via BLAST analyses of the mouse RefSeq RNA database using the GenBank partial-sequences referenced in [37]. Any PSG cDNA sequences that could still not be identified by this method were determined by BLAST analysis of the mouse genome database using known fragments of the sequence to be determined. This identified genomic contigs that could be interrogated for the 'missing' exonic sequences by pairwise BLAST analysis using the *Psg17 *cDNA sequence, or fragments thereof, as a probe. A similar procedure was applied to situations where the existence of alternatively spliced exons was suspected to reside within in a *Psg *gene-containing contig. In the cases where *Psg *mRNA sequences were built from genomic sequence, or splice variants were predicted, evidence for the existence of such mRNA species *in vivo *was tested by BLAST analysis using the mouse EST and Trace Archive EST databases.

### Bioinformatic analysis of the mouse PSG Ig domains

The coding sequences of the PSG domains were aligned using Clustal X [54]. For the production of rooted neighbour-joining (NJ) evolutionary trees, alignments were bootstrapped 1000 times. Evolutionary trees were constructed from the alignments using TreeView .

### Analysis of the *Psg32 *exon 1 sequence

Four hundred nanograms of DNA obtained from four inbred mouse strains (A/J, C57BL6/6J, YBR/Ei, and SWR/J) (Jackson Laboratory, Bar Harbor, Maine, USA) were amplified using the oligonucleotide primers 5'-AAGGAAGGACAGCAAAT and 5'- AGCTGTGAGCAGAAGAC (denaturation: 94°C, 30 s; annealing: 50°C, 30 s; extension: 72°C, 30 s; 30 cycles) with Pfu DNA polymerase (Stratagene) following the manufacturer's instructions. The 146 bp PCR products were subcloned into PCR-Script (Stratagene). Clones that hybridized to a *Psg32*-specific oligonucleotide, *Psg32*-exon 1, which binds to a sequence internal to the PCR primers were sequenced.

## Authors' contributions

ASM performed bioinformatics relating to *Psg *locus organisation, phylogenetic analyses, *Psg *expression studies, and drafted the manuscript. BF characterized *Psg *genomic clones and performed *Psg *expression studies. GD isolated the Psg BAC clones and analysed *Psg32 *exon 1 sequences in mouse strains. MB and FW contributed to *Psg *expression studies and YAC fragment assembly. TH and KO performed FISH analysis for identification of orientation of *Psg *subclusters. TM co-conceived the project and coordinated work performed in Cork. WZ co-conceived the project, coordinated all work performed in Germany, and performed bioinformatics relating to *Psg *phylogeny and expression.

## Supplementary Material

Additional File 1 **The gene sequence and predicted CDS and primary protein sequences for *Psg16 *(placental transcript), *Psg20*, *Psg24*, *Psg25*, *Psg26*, *Psg27*, *Psg29 and Psg31 ***A basic text file containing the primary genome data (with source HTGS or WGS information). Predicted CDS sequence is included along with translations. Reverse complement is abbreviated 'RC' in the text. Click here for file

Additional File 2 **Complete set of mouse *Psg *CDS sequences **A basic text file in FASTA format containing CDS sequences for all known mouse *Psgs *(not including the brain-specific variant of *Psg16*). Click here for file

Additional File 3 **Complete set of mouse PSG primary protein sequences **A basic text file in FASTA format containing primary protein sequences for all known mouse *Psgs *(not including the brain-specific variant of *Psg16*). Click here for file
